# Thermally stable and highly efficient red-emitting Eu^3+^-doped Cs_3_GdGe_3_O_9_ phosphors for WLEDs: non-concentration quenching and negative thermal expansion

**DOI:** 10.1038/s41377-021-00469-x

**Published:** 2021-02-01

**Authors:** Peipei Dang, Guogang Li, Xiaohan Yun, Qianqian Zhang, Dongjie Liu, Hongzhou Lian, Mengmeng Shang, Jun Lin

**Affiliations:** 1 State Key Laboratory of Rare Earth Resource Utilization, Changchun Institute of Applied Chemistry, Chinese Academy of Sciences, 130022 Changchun, China; 2 University of Science and Technology of China, 230026 Hefei, China; 3 Engineering Research Center of Nano-Geomaterials of Ministry of Education, Faculty of Materials Science and Chemistry, China University of Geosciences, 430074 Wuhan, China; 4 School of Material Science and Engineering, Shandong University, 266071 Jinan, China; 5 School of Applied Physics and Materials, Wuyi University, 529020 Guangdong, China

**Keywords:** Displays, Inorganic LEDs

## Abstract

Red phosphor materials play a key role in improving the lighting and backlit display quality of phosphor-converted white light-emitting diodes (pc-WLEDs). However, the development of a red phosphor with simultaneous high efficiency, excellent thermal stability and high colour purity is still a challenge. In this work, unique non-concentration quenching in solid-solution Cs_3_Gd_1 − *x*_Ge_3_O_9_:*x*Eu^3+^ (CGGO:*x*Eu^3+^) (*x* = 0.1–1.0) phosphors is successfully developed to achieve a highly efficient red-emitting Cs_3_EuGe_3_O_9_ (CEGO) phosphor. Under the optimal 464 nm blue light excitation, CEGO shows a strong red emission at 611 nm with a high colour purity of 95.07% and a high internal quantum efficiency of 94%. Impressively, this red-emitting CEGO phosphor exhibits a better thermal stability at higher temperatures (175–250 °C, >90%) than typical red K_2_SiF_6_:Mn^4+^ and Y_2_O_3_:Eu^3+^ phosphors, and has a remarkable volumetric negative thermal expansion (coefficient of thermal expansion, *α* = −5.06 × 10^−5^/°C, 25–250 °C). By employing this red CEGO phosphor, a fabricated pc-WLED emits warm white light with colour coordinates (0.364, 0.383), a high colour rendering index (CRI = 89.7), and a low colour coordinate temperature (CCT = 4508 K). These results indicate that this highly efficient red-emitting phosphor has great potential as a red component for pc-WLEDs, opening a new perspective for developing new phosphor materials.

## White LEDs: Warming up to shape-shifting phosphors

A luminescent material that undergoes an unusual heat-shrinking process shows promise for enhancing the efficiency of white light-emitting diodes (LEDs). Current technologies for generating white LEDs often combine near-ultraviolet light with background radiation from red, green, and blue phosphors. J.L. from China’s Changchun Institute of Applied Chemistry and colleagues have now developed a red phosphor that remains active at temperatures which shut down other red-light emitters. The team’s compound, a cesium-based metal oxide doped with rare earth ions, has a crystal structure that contracts upon heating and avoids symmetry-distorting effects associated with thermal quenching. This unique structure also supports higher dopant concentrations than typical phosphors for improved colour rendering in prototype white LEDs.

## Abstract (web summary)

A highly efficient red-emitting Cs_3_EuGe_3_O_9_ phosphor for high-performance pc-WLEDs is discovered with non-concentration quenching, favourable thermal stability, high colour purity and negative thermal expansion.

## Graphical abstract

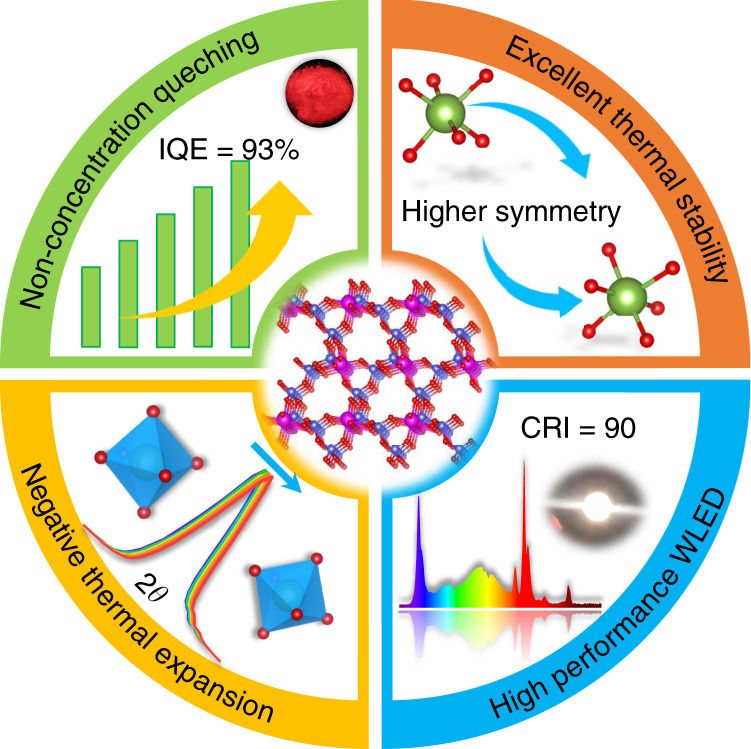

## Introduction

Phosphor-converted white light-emitting diodes (pc-WLEDs) have become the next-generation solid-state lighting source owing to their energy conservation, high efficiency, long durability and environmental friendliness^1–3^. The most common pc-WLEDs are fabricated by using two main methods: (1) combining a blue LED chip with a yellow phosphor and (2) combining a near-ultraviolet (n-UV) LED chip and tricolour (blue, green, red) phosphors^4,5^. However, regardless of the fabrication strategy, the development of red phosphors provides new opportunities for pc-WLEDs with both a high colour rendering index (CRI) and low colour coordinate temperature (CCT)^6^. To date, many highly efficient red phosphors have been developed based on versatile structural models, such as Eu^2+^-doped nitrides and Mn^4+^-doped fluorides^7,8^. Although many Eu^2+^-activated nitride phosphors, such as SrLiAl_3_N_4_:Eu^2+^ and Sr_2_Si_5_N_8_:Eu^2+^, present a high quantum efficiency (QE > 90%) and excellent thermal stability (150 °C, >90%), nitride raw materials are expensive, and their synthesis conditions are very harsh (high pressure, ≥2.5 MPa; high temperature ≥ 1500 °C)^9–11^. Typical Mn^4+^-doped fluorides, such as K_2_SiF_6_:Mn^4+^ and K_2_TiF_6_:Mn^4+^ phosphors, have a higher luminescence efficiency than Eu^2+^-doped nitrides, but there are two drawbacks, i.e. the use of massive HF acid and the low thermal stability, restricting their further application^12,13^. In addition to the above Eu^2+^-doped nitrides and Mn^4+^-doped fluorides, rare earth (RE) ion-activated oxides have also been extensively developed as tricolour phosphor materials due to their inexpensive raw materials and mild synthetic conditions^14–16^. At present, a great many RE ion-activated oxide blue or green phosphors have been developed, such as commercial green (Ba,Sr)_2_SiO_4_:Eu^2+^ and blue BaMgAl_10_O_17_:Eu^2+^ (BAM:Eu^2+^) phosphors with a high quantum efficiency (QE) and superb thermal stability^17–19^. Many RE ion-activated oxide red phosphors have also been investigated, especially Eu^3+^-doped oxide red phosphors with narrow bands and high colour purity^20,21^. For example, for commercial red Y_2_O_3_:Eu^3+^ phosphors, the QE is very high when excited under UV light^22^. However, this phosphor cannot be effectively excited by n-UV or blue light. Other developed Eu^3+^-doped oxides excited by n-UV or blue light have either a low QE or large thermal quenching^23,24^. Consequently, the design of new red-emitting phosphors excited by n-UV or blue light with low thermal quenching and a high QE under moderate synthesis conditions is a key challenge for emerging applications.

To obtain red phosphor with outstanding properties, Eu3+-doped red phosphors have attracted much interest. These phosphors have abundant transitions from the excited ^5^D_0_ level to the ^7^F_J_ (*J* = 0, 1, 2, 3, 4) levels of the ^4^f_6_ configuration, and their emission properties are determined by the local environment of Eu^3+^-occupied sites in the host lattice^25^. The main emissions of Eu^3+^ come from the ^5^D_0_ → ^7^F_1_ and ^5^D_0_ → ^7^F_2_ transitions, peaking in the orange (585–600 nm) and red (610–630 nm) spectral regions, respectively. According to the Judd-Ofelt theory, the electric dipole transition (^5^D_0_ → ^7^F_2_) strength of the parity prohibition is much stronger than the magnetic dipole transition (^5^D_0_ → ^7^F_1_) allowed by the parity, indicating that the Eu^3+^ is in a non-symmetric lattice position^26^. When Eu^3+^ occupies sites with non-inversion symmetry, a narrow-band red emission with high colour purity at ~610–620 nm can be easily achieved. However, the weak and narrow absorption in the n-UV and blue regions in most Eu^3+^-doped phosphors due to the parity-forbidden transitions (^7^F_0_ → ^5^D_4_, ^5^L_6_, ^5^D_3_) leads to a low QE, which and renders their application in n-UV-based pc-WLEDs difficult^27^. Therefore, it is necessary to enhance the absorption in the n-UV and blue regions of Eu^3+^-doped red phosphors to further improve the QE for expanding applications. Accordingly, the identification of suitable hosts with a compact structure and non-inversion symmetry sites occupied by Eu^3+^ should be first taken into account when exploring Eu^3+^-doped phosphors. Many Eu^3+^-based red phosphors have been investigated, such as Eu^3+^-based borates (e.g. LaSc_3_(BO_3_)_4_:Eu^3+^), pyrophosphates (e.g. MgIn_2_P_4_O_14_:Eu^3+^) and silicates (e.g. Y_2_Mg_2_Al_2_Si_2_O_12_:Eu^3+^)^28–30^. However, germanates are less studied, although they offer the same potential applications^31^. Among various germanate systems, new caesium RE germanate Cs_3_GdGe_3_O_9_ is attracting considerable attention. In 2019, Morrison et al. reported that Cs_3_GdGe_3_O_9_, which crystallises in an orthorhombic system with space group Pna21, has good magnetic properties^32^. As reported, there is one Gd^3+^ site (coordination number, CN = 6) with non-inversion symmetry in this host lattice. This cation site in the Cs_3_GdGe_3_O_9_ host should be suitable for the doping of Eu^3+^ ions to obtain narrow-band red emission. To date, the optical properties of different concentrations of Eu^3+^-doped Cs_3_GdGe_3_O_9_ have not been investigated and reported in detail.

In addition, the luminescence stability is closely related to the structural stability, and the stronger the structural rigidity is, the better the thermal stability is^33^. Most phosphor materials expand with increasing temperature, which is commonly described as positive thermal expansion (PTE)^34,35^. A large PTE can result in thermal stress and even material failure. Nevertheless, to date, few negative thermal expansion (NTE) phosphor materials that exhibit shrinkage volumes in a certain temperature range have been investigated^36^. This volume contraction can be caused by the transverse vibration of corner-sharing atoms in frameworks with strong structural rigidity composed of polyhedra^37^. Intriguingly, materials with an NTE can withstand large thermal gradients and are less subject to failure due to their potential high thermal shock fracture resistance when exposed to temperature extremes^38^. Therefore, the luminescence of NTE phosphor materials with strong structural rigidity is expected to still maintain high efficiency at high temperatures (>200 °C). Therefore, it is meaningful to explore this kind of phosphor material with an NTE to improve the luminescence stability and efficiency.

Herein, we reported a non-concentration quenching red Cs_3_EuGe_3_O_9_ (CEGO) phosphor with remarkable NTE properties. Under n-UV or blue light excitation, this phosphor exhibits a highly efficient red emission at 611 nm and high internal quantum efficiency (IQE = 94%). Meanwhile, CEGO shows good thermal stability, and the luminescence intensity can maintain ~90% of the initial value at 250 °C. A pc-WLED device is constructed using red CEGO combined with commercial blue and green phosphors, giving CIE colour coordinates of (0.364, 0.383), a low CCT = 4508 K and a high CRI = 89.7. This work represents an important step towards achieving highly efficient and thermally stable red emission for solid-state illumination and backlit displays.

## Results

### Crystal structure and phase identification

**Fig. 1:**
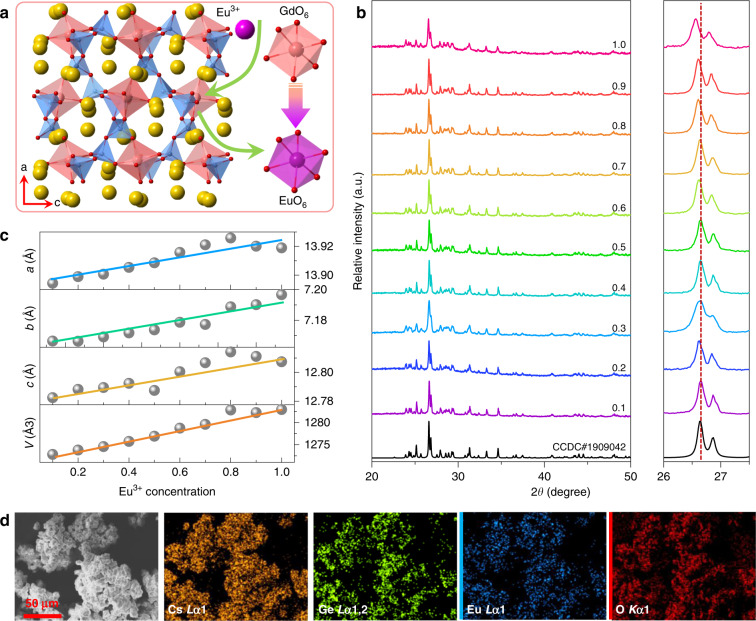
Crystal structure and micromorphology. **a** Crystal structure of the Cs_3_GdGe_3_O_9_ host compound (colour balls: Cs yellow, Gd light red, Ge light blue, O red). **b** XRD patterns (2*θ* = 20–60°) and enlarged XRD patterns at 2*θ* = 26–27.5° of CGGO:*x*Eu^3+^ (*x* = 0.1–1.0) samples. **c** The variation in cell parameters (**a**, **b**, **c**, V) for CGGO:*x*Eu^3+^ (*x* = 0.1–1.0) samples. **d** Elemental mapping images of the CEGO sample

The Cs_3_GdGe_3_O_9_ (CGGO) compound crystallises in an orthorhombic system with space group Pna21. The unit of this compound contains three Cs sites, one Gd site, three Ge sites, and nine O sites. As shown in Fig. 1a, Gd^3+^ is coordinated by six O atoms to form a [GdO_6_] octahedron, and the Ge^4+^ ions are tetrahedrally coordinated by four O atoms. The GeO_4_ tetrahedra form zigzag chains with a Ge_6_O_18_ repeating unit by corner sharing. These zigzag chains are connected into a frame via GdO_6_ octahedra, which share corners with two GeO_4_ tetrahedra each from three chains. The Cs atoms occupy voids within this structure. Figure 1b demonstrates the X-ray diffraction (XRD) patterns of Cs_3_Gd_1 − *x*_Ge_3_O_9_:*x*Eu^3+^ (CGGO:*x*Eu^3+^) (*x* = 0.1–1.0) solid-solution samples. Obviously, all diffraction peaks of the studied samples show a good correlation with the standard card (CCDC#1909042), even at the highest Eu^3+^ doping concentrations (*x* = 1.0). As the Eu^3+^ concentration increases from 0.1 to 1.0, a small shift in the lower angle direction in the diffraction peaks (2*θ* = 26–27.5°) is observed, indicating the successful incorporation of Eu^3+^. The lattice expansion is ascribed to the occupation of the Gd^3+^ (CN = 6, *r* = 0.938 Å) site by Eu^3+^ (CN = 6, *r* = 1.17 Å). The XRD data Rietveld profile refinements are used to characterise the microstructure evolution of CGGO:*x*Eu^3+^ (*x* = 0.1–1.0).

Figure S1 shows the refinement XRD patterns of representative CGGO:*x*Eu^3+^ (*x* = 0.1, 0.5, 0.9, 1.0) samples. The cell parameters and accredited R-factors of all samples imply the crystallisation of the solid-solution (Table S1). These samples crystallise in an orthorhombic phase with space group Pna21 (33). The refined lattice parameters are in the following ranges: *a* = 13.8944(19)–13.9258(9) Å, *b* = 7.1665(10)–7.1967(11) Å, *c* = 12.7825(18)–12.8142(8) Å and *V* = 1272.81(31)–1282.93(31) Å^3^. The detailed atomic coordinates of representative CGGO:*x*Eu^3+^ (*x* = 0.1, 0.5, 0.9, 1.0) are listed in Table S2. There is one Gd^3+^ site for Eu^3+^ doping. These results further confirm the phase purity of the as-prepared samples and verify that Eu^3+^ is successfully doped into the CGGO crystal and completely replaces Gd^3+^ to transform into CEGO. Figure 1c presents the lattice parameter (*a*, *b*, *c*) and volume (*V*) as a function of Eu^3+^ content. It is clear that the cell parameter almost linearly increases as the Eu^3+^ content increases from 0.1 to 1.0 and follows Vegard’s law^39^. Figure 1d shows the morphology and elemental distribution of CEGO, which displays a slight agglomeration of these phosphor particles. The elemental mapping images indicate that Cs, Eu, Ge and O are homogeneously distributed in CEGO. This result indicates the successful synthesis of the designed CGGO:Eu^3+^ phosphors.

### PL properties of the CGGO:Eu^3+^ phosphors

**Fig. 2:**
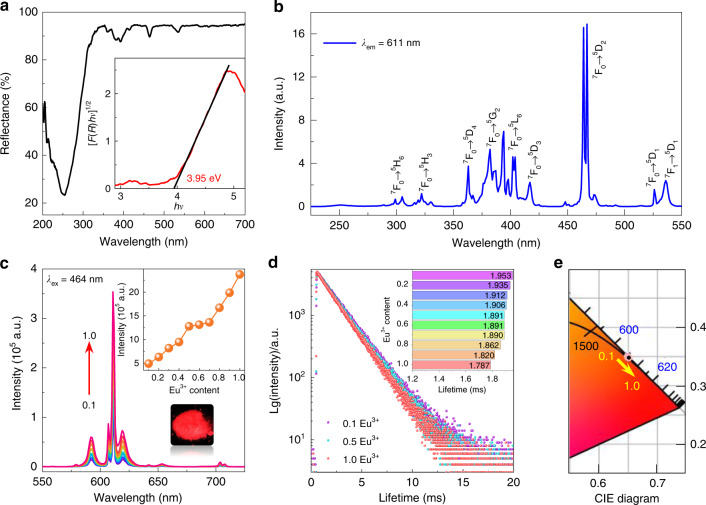
Photoluminescence properties. **a** Diffuse reflection spectrum of CEGO. The inset depicts the relationship of [*F*(*R*)*hv*]^1/2^ versus *hv*. **b** PLE spectrum of CEGO monitored at a 611 nm emission wavelength. **c** PL spectra of CGGO:*x*Eu^3+^ (*x* = 0.1–1.0) samples excited at 464 nm blue light. The inset shows the PL intensity of CGGO:*x*Eu^3+^ (*x* = 0.1–1.0) samples. **d** PL decay curves of the CGGO:*x*Eu^3+^ (*x* = 0.1–1.0) samples excited at 464 nm and monitored at 611 nm wavelength. **e** CIE chromaticity coordinate diagram of the CGGO:*x*Eu^3+^ (*x* = 0.1–1.0) samples

Figure 2a depicts the diffuse reflectance (DR) spectrum of CEGO in the UV–vis region. Two absorption peaks at 393 and 464 nm from the ^7^F_0_–^5^L_6_ and ^7^F_0_–^5^D_2_ transitions of Eu^3+^ ions are observed, indicating that CEGO can match well with the n-UV or blue LED chips. The optical band gap (*E*_g_) is calculated by using the Kubelka–Munk equation^40^:

1 $$\begin{array}{l}\left[ {F\left( R \right)h\nu } \right]^{1/2} \,=\, A\left( {h\nu \,-\, E_g} \right)\\ F\left( R \right) \,=\, \left( {1 \,-\, R} \right)^2/2R\end{array}$$

where *R* is the measured DR coefficient (%), *F*(*R*) represents the absorption, *hν* is the photon energy and *A* represents the absorption constant. The inset of Fig. 2a displays the plots of [*F*(*R*)*hν*]^1/2^ versus 1240/*λ* of CEGO. The *E*_g_ of CEGO is calculated to be 3.95 eV. This illustrates that CEGO with an appropriate *E*_g_ is suitable for n-UV- or blue-based pc-WLED devices.

Figure 2b depicts the PLE spectrum of CEGO monitoring the characteristic ^5^D_0_–^7^F_2_ emission (611 nm) of Eu^3+^ ions. The PLE spectrum contains a very weak charge transfer band (CTB) and a series of sharp peaks. The CTB located at 225–275 nm with a maximum value at 260 nm can be attributed to the charge transfer (Eu^3+^–O^2−^) transition generated by electron transfer from the ligand O^2−^ (2p^6^) orbital to the empty state of 4f^6^ of Eu^3+^. The 2p electrons of O^2−^ are tightly bound to Ge^4+^ because of the high charge of Ge^4+^, which leads to a weak CTB of Eu^3+^ in CEGO. As previously discussed, the [EuO_6_] octahedra connect the [Ge_6_O_18_] units with zigzag chains via corner sharing to form the framework of CEGO. Eu^3+^ and Ge^4+^ are regularly distributed in the two positions in CEGO. Ge^4+^ and Eu^3+^ compete with each other for the electron cloud of O^2−^ via their Coulombic potential (φ). The Coulombic potential can be expressed by the following formula^41^: 

2 $$\phi \,=\, Ze^2/r$$

where *Z* and *e* are the charges of the cation and electron, respectively, and *r* is the ionic radius. The higher *Z* value and smaller *r* value of Ge^4+^ compared with those of Eu^3+^ result in a higher Coulombic potential. This allows the augmentation of the overlap of the electron clouds between O^2−^ and Ge^4+^, illustrating stronger O^2−^–Ge^4+^ covalent bonds and reducing the mobilities from O^2−^ to Eu^3+^. Accordingly, the CTB of O^2−^–Eu^3+^ in CEGO is weak. In addition, the sharp peaks originate from the 4f–4f transitions of Eu^3+^, which are located at 304, 322, 362, 382, 393–403, 416 and 464 nm and belong to the ^7^F_0_ → ^5^H_6_, ^7^F_0_ → ^5^H_3_, ^7^F_0_ → ^5^D_4_, ^7^F_0_ → ^5^G_2_, ^7^F_0_ → ^5^L_6_, ^7^F_0_ → ^5^D_3_, and ^7^F_0_ → ^5^D_2_ transitions, respectively. Obviously, some strong peaks from 370 to 410 nm and the strongest peak at 464 nm indicate that this red-emitting CEGO phosphor matches well with n-UV and blue chips for application in pc-WLEDs.

Figure 2c depicts the PL spectra of CGGO:*x*Eu^3+^ (*x* = 0.1–1.0) phosphors under 464 nm blue light excitation at room temperature. The sharp emission peaks at ~592 and 611 nm correspond to ^5^D_0_ → ^7^F_1_ and ^5^D_0_ → ^7^F_2_, respectively. The dominate red emission at 611 nm is attributed to the electric dipole transition ^5^D_0_ → ^7^F_2_, indicating that Eu^3+^ is located at a position with non-inversion symmetry according to the Judd-Ofelt theory. This result is in agreement with the crystal structure where Eu^3+^ takes on the Gd^3+^ site in CGGO without inversion symmetry. Interestingly, the PL intensity gradually increases as the Eu^3+^ content increases from 0.1 to 1.0, without concentration quenching (inset of Fig. 2c). Generally, concentration quenching occurs at a low doping content in many Eu^3+^-doped phosphors. For instance, the quenching concentration of commercial red Y_2_O_3_:Eu^3+^ phosphor is found to be 0.05. Concentration quenching of the emission is attributed to the increased probability of energy migration between the luminescent centres. Energy transfer can be radiative (so-called photon exchange) or nonradioactive (due to short-range exchange interactions or long-range multipolar interactions)^27^. Thus, concentration quenching means that the distance between the luminescent centres becomes shorter with increasing activator concentration. Blasse noted that the exchange interaction between Eu^3+^ ions is generally responsible for the energy transfer when the distance between Eu^3+^ ions is less than or equal to 5 Å^42^. Usually, the Eu–Eu distance is larger than 5 Å in Eu^3+^-doped phosphors, exchange interactions become ineffective, only multipolar interactions can be of importance, and they will be weak nevertheless^43^. In the structure of CGGO, the shortest distance between two Gd^3+^ ions is up to 6.836 Å, which is larger than the nearest Eu^3+^ distance in other reported zero concentration quenching phosphors, such as LaSc_3_(BO_3_)_4_:Eu^3+^ (6.22 Å)^26^, Ba_6_Gd_2_Ti_4_O_17_:Eu^3+^ (5.93 Å)^21^, and K_5_Y(P_2_O_7_)_2_:Eu^3+^ (5.6 Å)^44^. The longer the distance between nearest-neighbour Eu^3+^ ions is, the more favourable for Eu^3+^ doping is at a high concentration. Moreover, this distance becomes longer with increasing Eu^3+^ concentration (Fig. S2). These results imply that the distance is long enough to favour Eu^3+^ emission and weaken the energy transfer.

Figure 2d displays the PL lifetime decay curves of the CGGO:*x*Eu^3+^ (*x* = 0.1–1.0) phosphors (*λ*_ex_ = 464 nm, *λ*_em_ = 611 nm) at room temperature. All the decay curves are well fitted by a mono-exponential formula^45^:

3 $$I(t) \,=\, I_0 \,+\, A\exp \left( { - t/\tau } \right)$$

where *I*(*t*) is the corresponding PL intensity at time *t*, *I*_0_ is the initial PL intensity, *A* represents a constant, and *τ* is the PL lifetime. The PL lifetimes were calculated to be 1.953–1.787 ms for the CGGO:*x*Eu^3+^ (*x* = 0.1–1.0) phosphors as the Eu^3+^ concentration increased from 0.1 to 1.0, respectively. Such close PL lifetimes suggest the possibility of non-concentration quenching. This result is different from the other Eu^3+^-doped phosphors with concentration quenching, where the PL lifetime decreases sharply when quenching occurs.

**Fig. 3:**
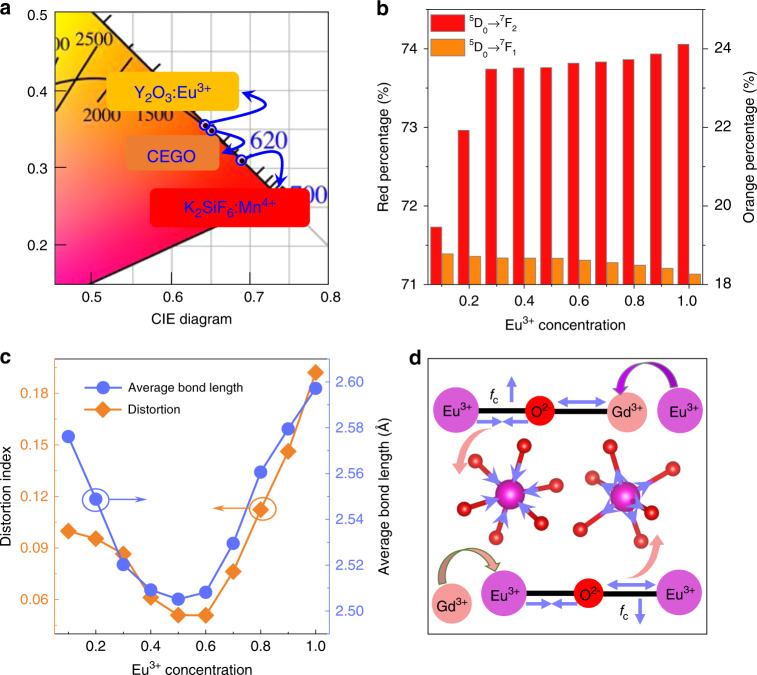
Red-to-orange ratio and symmetry. **a** CIE chromaticity coordinate diagram of CEGO compared with the commercial red Y_2_O_3_:Eu^3+^ and K_2_SiF_6_:Mn^4+^ phosphors. **b** The variation trend of the ^5^D_0_–^7^F_2_ (red) and ^5^D_0_–^7^F_1_ (orange) transitions in the PL spectra for the CGGO:*x*Eu^3+^ (*x* = 0.1–1.0) samples. **c** Variation in the distortion of the EuO_6_ octahedron and average bond length of Gd/Eu–O with increasing Eu^3+^ concentration in the CGGO:*x*Eu^3+^ (*x* = 0.1–1.0) samples. **d** Schematic diagram of the variation in the covalence degree and length of Eu–O bonds in the Eu^3+^-doped CGGO crystal structure

The CIE chromaticity coordinates and colour purities of CGGO:*x*Eu^3+^ (*x* = 0.1–1.0) were calculated and are listed in Fig. 2e and Table S3. The colour purities were calculated as follows^46^:

4 $${\mathrm{Colour}}\,{\mathrm{purity}} \,=\, \frac{{\sqrt {\left( {{{x \,-\, x}}_{\mathrm{i}}} \right)^{\mathrm{2}} \,+\, \left( {{{y}} \,-\, {{y}}_{\mathrm{i}}} \right)^2} }}{{\sqrt {\left( {{{x}}_{\mathrm{d}} \,-\, {{x}}_{\mathrm{i}}} \right)^2 \,+\, \left( {{{y}}_{\mathrm{d}} \,-\, {{y}}_{\mathrm{i}}} \right)^2} }}$$

where (*x*, *y*), (*x*_i_, *y*_i_) and (*x*_d_, *y*_d_) represent the coordinates, dominant wavelength of the studied samples, and white illumination, respectively. For CEGO, the colour purity is as high as 95.07%, which is higher than that of commercial red Y_2_O_3_:Eu^3+^ and K_2_SiF_6_:Mn^4+^ phosphors. The CIE chromaticity coordinates of CEGO can reach (0.6517, 0.348), and the corresponding location is marked in the CIE diagram compared with commercial red phosphors, as displayed in Fig. 3a. The IQEs and external QEs (EQEs) of CGGO:*x*Eu^3+^ (*x* = 0.1–1.0) were measured and are listed in Table S3. The IQE exhibits a gradual increase as the Eu^3+^ content increases, which is consistent with the corresponding PL spectra discussed previously. The increase in the IQE can be attributed to the reduction in the energy loss caused by non-radiative transitions. The IQE of CEGO (~94%) is much higher than that of the commercial red Y_2_O_3_:Eu^3+^ phosphor (~8%) under 464 nm light excitation and almost the same as the IQE value (~95%) of another commercial red K_2_SiF_6_:Mn^4+^ phosphor. However, the EQE values are relatively lower than K_2_SiF_6_:Mn^4+^ due to the narrow absorption at 464 nm.

In addition, the Eu^3+^ doping concentration influences the red-to-orange ratio (*R*/*O*) of Eu^3+^ emission, which is the ratio of the integral intensity of 600–630 nm (red, ^5^D_0_ − ^7^F_2_) to the integral intensity of 575–600 nm (orange, ^5^D_0_ − ^7^F_1_). The relative contribution of the ^5^D_0_ − ^7^F_2_ transition (red emission, at 611 nm) increases with increasing Eu^3+^ content, while that of the ^5^D_0_ − ^7^F_1_ transition (orange emission, at 592 nm) has a decreasing trend, as demonstrated in Fig. 3b. The red percentage is the ratio of the integral intensity of 600–630 nm to the integral intensity of 550–750 nm, while the orange percentage is the ratio of the integral intensity of 575–600 nm to the integral intensity of 550–750 nm. It is known that the electric dipole transition ^5^D_0_–^7^F_2_ is hypersensitive to site symmetry, while the magnetic dipole transition ^5^D_0_-^7^F_1_ is insensitive. The electric dipole transition ^5^D_0_–^7^F_2_ is predominant, as Eu^3+^ is located at sites with non-inversion symmetry; thus, the red-to-orange ratio is used to reflect the symmetry of the site occupied by Eu^3+^. It can be seen that the *R*/*O* increases as the Eu^3+^ content (*x*) increases, revealing that introducing Eu^3+^ ions could perturb the symmetry of the site. To investigate the symmetry of the [EuO_6_] octahedron when Eu^3+^ is doped into the host lattice, the octahedral distortion can be estimated as follows^47^:

5 $${{D}} \,=\, \frac{1}{{{n}}}\mathop {\sum}\limits_{{{i}}={\mathrm{1}}}^{{n}} {\frac{{\left| {{{l}}_{\mathrm{i}} \,-\, {{l}}_{{\mathrm{av}}}} \right|}}{{{{l}}_{{\mathrm{av}}}}}}$$

where *l*_i_ is the bond length between the central cation and the ith coordination ligand and *l*_av_ is the average bond length. The average bond length of Gd/Eu–O and distortion index of [EuO_6_] can be calculated according to the Rietveld refinement results, as presented in Fig. 3c. Theoretically, a larger distortion index results in lower symmetry, leading to a larger R/O. However, the calculated results are inconsistent with the experimental results when *x* ≤ 0.5. The distortion index decreases, and the symmetry of the [EuO_6_] octahedron increases with increasing Eu^3+^ content (*x*), but the *R*/*O* still increases as the Eu^3+^ content increases from 0.1 to 0.5. In fact, in addition to the symmetry of the [EuO_6_] octahedron, the covalence degree (f_c_) of the Eu–O bonds can also affect the ^5^D_0_–^7^F_2_ transition intensity by further breaking the parity selection rules^48^. A schematic illustration of the variation in the covalence degree and Eu–O bond length is proposed in Fig. 3d. At lower doping concentrations (*x* ≤ 0.5), the larger Eu^3+^ ions occupy the smaller Gd^3+^ sites, leading to a longer Gd–O bond length. In terms of the competition between the Eu–O bonds and their neighbouring Gd–O bonds, the decreasing attraction of Gd^3+^ ions means that the Eu–O bonds become more compact and shorter. Thus, the covalence degree of Eu–O bonds increases as the Eu^3+^ content increases from 0.1 to 0.5, illustrating that the ^5^D_0_–^7^F_2_ transition of Eu^3+^ is more determined by the covalence degree of Eu–O bonds when *x* ≤ 0.5. At higher doping concentrations (*x* > 0.5) in this system, Gd^3+^ can replace Eu^3+^, leading to longer neighbouring Eu–O bonds. The covalence degree of Eu–O bonds decreases as the Eu^3+^ concentration increases from 0.5 to 1.0. At this point, the ^5^D_0_–^7^F_2_ transition of Eu^3+^ depends more on the lattice distortion of the [EuO_6_] polyhedron when *x* > 0.5. The distortion index of the [EuO_6_] polyhedron increases, and the symmetry of the [EuO_6_] polyhedron decreases with increasing Eu^3+^ concentration (*x* > 0.5), leading to an increase in *R*/*O*. Under the combined effect of lattice distortion and covalence degree factors, the PL intensity of Eu^3+^ (611 nm) from the electric dipole transition is enhanced, and *R*/*O* increases with increasing Eu^3+^ concentration. This change leads to the continuous enhancement of the overall PL intensity and non-concentration quenching.

### Temperature-dependent PL properties

**Fig. 4:**
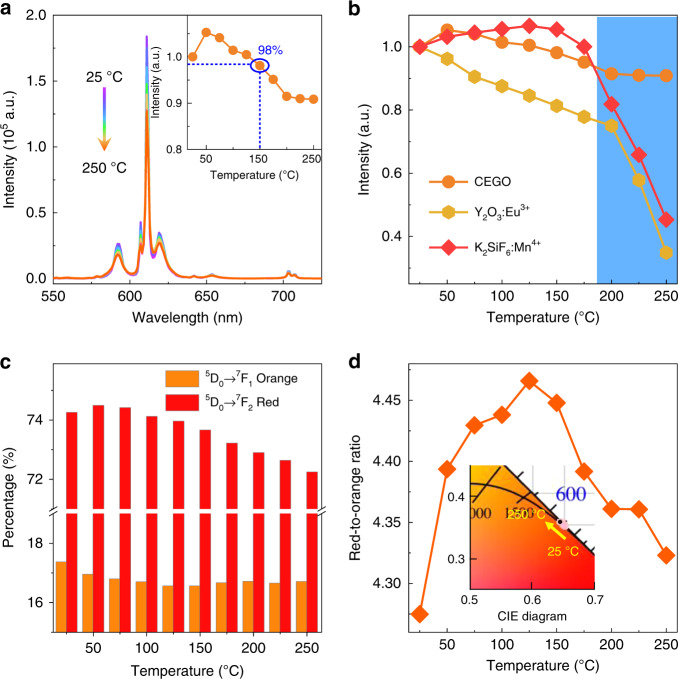
Temperature-dependent photoluminescence properties of CEGO. **a** Temperature-dependent PL spectra of CEGO from 25 to 250 °C. The PL intensity of CEGO is depicted in the inset. **b** Temperature-dependent PL intensity of CEGO compared with the commercial red Y_2_O_3_:Eu^3+^ and K_2_SiF_6_:Mn^4+^ phosphors. **c** The variation trend of the ^5^D_0_–^7^F_2_ (red) and ^5^D_0_–^7^F_1_ (orange) transitions in temperature-dependent PL spectra for CEGO from 25 to 250 °C. **d** The variation in the *R*/*O* ratio for CEGO from 25 to 250 °C. The CIE chromaticity coordinate diagram of CEGO at different temperatures (25–250 °C) is shown in the inset

Figure 4a depicts the temperature-dependent PL spectra of the red CEGO phosphor excited at 464 nm blue light at different temperatures (*T* = 25–250 °C). All peak positions are unchanged, and the PL intensity rarely decreases with increasing temperatures from 25 to 250 °C due to an enhancement in the probability of non-radiative transitions. This finding is attributed to the intensified thermal vibration of the matrix lattice in the high-temperature environment, the increased thermally activated phonons and the strengthened interaction between electrons and phonons. The PL intensity of CEGO remains at 98% of the initial value at 150 °C (inset of Fig. 4a), which is better than that of the commercial red Y_2_O_3_:Eu^3+^ phosphor and slightly less than that of another commercial K_2_SiF_6_:Mn^4+^ phosphor (Fig. 4b). Impressively, CEGO has good thermal stability in the high-temperature region (200–250 °C), and its PL intensity is decreased by 10% of the initial intensity (25 °C), which is much better than that of the commercial Y_2_O_3_:Eu^3+^ and K_2_SiF_6_:Mn^4+^ phosphors. The temperature-dependent PL intensity of CGGO:*x*Eu^3+^ (*x* = 0.1–1.0) is displayed in Fig. S3. As the Eu^3+^ concentration increases from 0.1 to 1.0, the thermal quenching becomes weaker, and the PL intensity at 250 °C exhibits an increasing trend (inset of Fig. S3) due to the stronger structural rigidity. It is known that the increased probability of non-radiative transition will lead to a shortened PL lifetime. The PL decay lifetimes of CGGO:*x*Eu^3+^ (*x* = 0.1, 0.5, 0.9, 1.0) as a function of temperature (25–250 °C) are shown in Fig. S4. The decay curves at different temperatures seriously overlap, implying that the PL lifetimes hardly change with increasing temperature. In addition, the PL lifetime of CEGO decreases slightly with increasing temperature, and increases at approximately 175 °C, indicating the good thermal stability of CEGO at 175–250 °C. As discussed previously, the intensity ratio of the red (^5^D_0_–^7^F_2_) to orange (^5^D_0_–^7^F_1_) emission is used to reflect the symmetry of the sites. The variation in the red and orange percentages of CEGO at different temperatures (25–250 °C) is depicted in Fig. 4c. The *R*/*O* increases when *T* < 150 °C but decreases at *T* > 150 °C, as shown in Fig. 4d, indicating that the symmetry of the [EuO_6_] polyhedron first decreases and then increases with increasing temperature. The CIE chromaticity coordinates and colour purities of the CEGO phosphor at various temperatures are calculated, as given in the inset of Fig. 4d and listed in Table S4. The colour stability and slight decrease in colour purity also indicate the good thermal stability of CEGO.

**Fig. 5:**
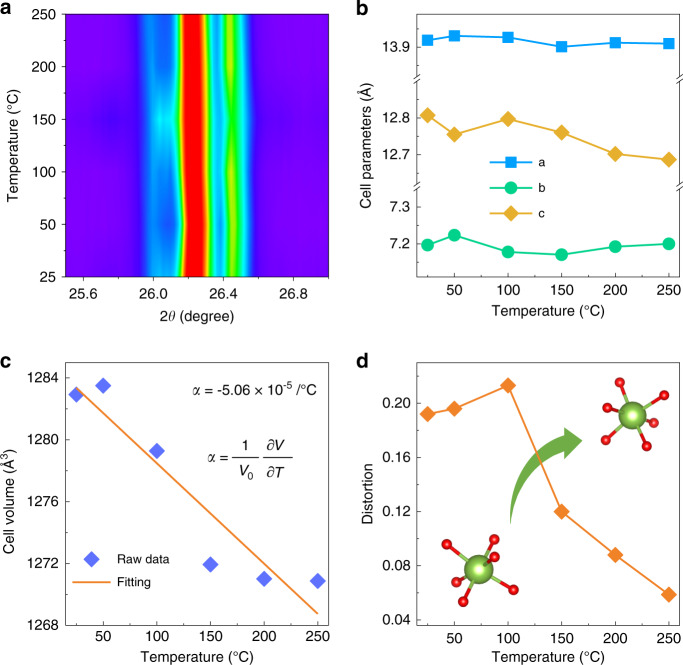
Negative thermal expansion. **a** Enlarged XRD patterns (2*θ* = 25.5–27°) of CEGO at different temperatures (25–250 °C). **b** The variation in cell parameters (**a**–**c**) for CEGO from 25 to 250 °C. **c** The linear fitting of cell volumes over the temperature range from 25 to 250 °C. **d** The variation in the distortion of the EuO_6_ octahedron with increasing temperature from 25 to 250 °C

To investigate the relationship between the lattice structure and PL properties of the red CEGO phosphor at different temperatures, temperature-dependent XRD was performed. Figure S5 illustrates the temperature-dependent XRD patterns (2*θ* = 20–50°) of CEGO at different temperatures (25–250 °C). The CEGO still maintains a pure phase at various temperatures, indicating that the CEGO has good thermal stability from room temperature to 250 °C. The enlarged XRD patterns at 2*θ* = 25.5–27° are given in Fig. 5a, and all the diffraction peaks do not shift to a lower angle, which means that this CEGO has a zero or negative thermal expansion (ZTE or NTE) performance. The variation in the cell parameters (*a*, *b*, *c*) of CEGO with increasing temperature from 25 to 250 °C according to Rietveld refinements is displayed in Fig. 5b. The cell parameters *a* and *b* show a slight change at various temperatures, while *c* exhibits a decreasing trend. The cell volume (V) decreases obviously with increasing temperature (Fig. 5c), demonstrating that volumetric NTE in CEGO occurs with increasing temperature. The bulk thermal expansion coefficient (TEC) is evaluated using the following formula^49^:

6 $$\alpha \,=\, \frac{1}{{V_0}}\frac{{\partial V}}{{\partial T}}$$

where α is the bulk TEC, *V* is the cell volume, and *T* is the temperature. The bulk TEC of this NTE phosphor was calculated to be −5.06 × 10^−5^/°C from the cell volumes. Indeed, the orthorhombic structure of this CEGO has polyhedral connectivity and consists of corner-sharing semirigid EuO_6_ octahedra and GeO_4_ tetrahedra. The Eu–O-Ge linkages between polyhedra undergo thermally excited transverse vibrations, causing cooperative rotations of the polyhedra and leading to a decrease in the unit cell volume as the temperature increases. Thus, this red CEGO phosphor with an orthorhombic structure can be regarded as a NTE material in the elevated temperature range. This strong structural rigidity leads to little thermal quenching.

According to the previous discussion, a smaller distortion index can result in the higher symmetry, leading to a smaller *R*/*O* ratio. To investigate the symmetry of the [EuO_6_] octahedron in this structure under different temperatures (*T* = 25–250 °C), the distortion indexes of the [EuO_6_] octahedron were calculated by equation (5), as given in Fig. 5d. The distortion index becomes larger when the temperature increases from 25 to 100 °C, while the distortion index decreases with increasing temperature from 150 to 250 °C. This result reveals that the symmetry of the [EuO_6_] octahedron becomes lower when *T* < 150 °C and higher when *T* > 150 °C, which is consistent with the change in the *R*/*O* of CEGO at different temperatures.

**Fig. 6:**
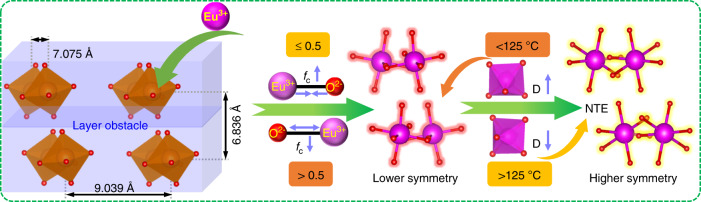
Relationship between structural feature and photoluminescence properties. Schematic illustration of the effect of the crystal structure on the non-concentration quenching and temperature-dependent PL properties

In brief, the structural features have a great effect on the PL properties. A schematic illustration of the relationship between the structural feature and the non-concentration quenching and temperature-dependent PL properties is illustrated in Fig. 6. In this structure of CGGO, the shortest distance between two Gd^3+^ ions on the same layer is 7.075 Å, and the shortest distance of Gd^3+^ ions on two neighbouring layers is 6.836 Å. The detailed structure of the layer-distributed Gd sites is displayed in Fig. S6. It should be mentioned that the ‘layer structure’ used for Cs_3_GdGe_3_O_9_ is only a man-made description, which is usually used for other phosphors, such as K_5_Y(P_2_O_7_)_2_ and Ba_6_Gd_2_Ti_4_O_17_^21,44^. These phosphors have a common feature, whereby some atomic groups are stacked in layers, and the interlayers are filled with large alkaline or alkaline-earth ions. Then, the binding force for these layers is just the weak interionic force between the alkaline or alkaline-earth ions and oxygen ions. In the CGGO structure, the Gd^3+^ ions are separated by the Cs^+^ ions layer by layer along the c-axis. The inter- and intralayer distances between Gd^3+^ ions are long enough to favour Eu^3+^ emission while weakening the energy transfer between them with increasing Eu^3+^ concentration, which leads to non-concentration quenching. Moreover, such a layer-built CGGO host lattice could provide a layer obstacle to restrict the interlayer energy migration of Eu^3+^ ions, and then greatly reduce the possibility of the quenching centres capturing the effective energy, even at a Eu^3+^ concentration of 100%. Under the combined effect of lattice symmetry and covalence degree factors, the *R*/*O* ratio shows a continuous enhancement with increasing Eu^3+^ concentration, and the highly efficient red emission of CEGO is realised. With regard to the thermal stability of CEGO, the smaller lattice distortion of the [EuO_6_] octahedron leads to the higher symmetry of [EuO_6_] in the high-temperature region (>150 °C). The thermal stability is related to the lattice symmetry, and a higher symmetry of the lattice means a better thermal stability. Consequently, this non-concentration quenching red phosphor has good thermal stability in the high-temperature region.

Considering that both QE and thermal stability are key factors for evaluating the performance of phosphors for applications, a comparison of the as-prepared CEGO phosphor with some reported Eu^3+^-doped phosphors and typically commercial red phosphors is summarised in Table S5. The PL performance of CEGO is superior to those of Y_2_O_3_:Eu^3+^ and almost catches up with K_2_SiF_6_:Mn^4+^ ^22,50^.

### Application of CEGO in pc-WLEDs

**Fig. 7:**
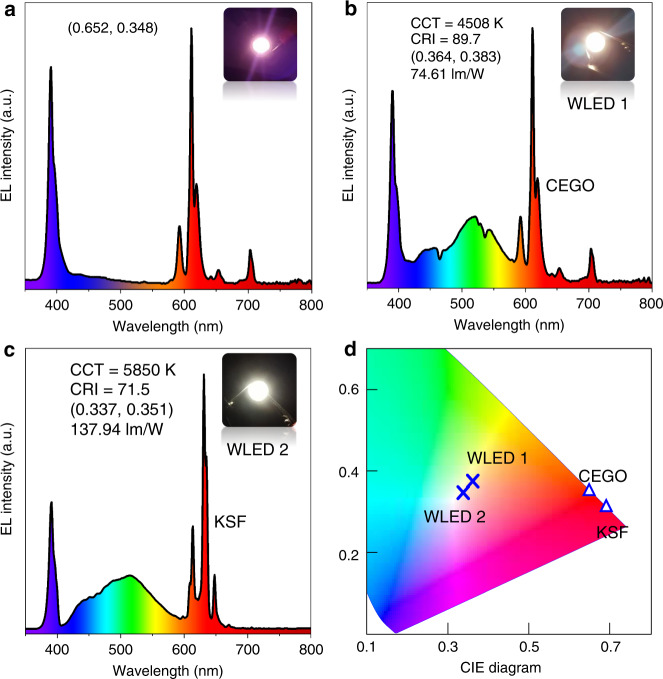
pc-WLEDs applications for CEGO. EL spectra and device photos of (**a**) CEGO + n-UV chip, (**b**) pc-WLED-1 device (n-UV chip + blue BAM:Eu^2+^ + green (BaSr)_2_SiO_4_:Eu^2+^ + red CEGO) and (**c**) pc-WLED-2 (n-UV chip + blue BAM:Eu^2+^ + green (BaSr)_2_SiO_4_:Eu^2+^ + red K_2_SiF_6_:Mn^4+^). **d** CIE chromaticity coordinate diagram of two pc-WLED devices and red phosphors

The preliminary application of this highly efficient red CEGO phosphor is demonstrated in Fig. 7. This phosphor can be directly excited by a n-UV LED chip and converts the light from the chip into bright and pure red emission (Fig. 7a). For fabrication, the blue light excitation band of CEGO is narrow and needs to be further improved. n-UV chips were used to fabricate the pc-WLEDs. Additionally, a warm pc-WLED-1 (Fig. 7b) was fabricated by the red CEGO, green (Ba,Sr)_2_SiO_4_:Eu^2+^, and blue BAM:Eu^2+^ phosphors together with a 395 nm n-UV LED chip. The performance of this pc-WLED-1 with the CIE chromaticity coordinates of (0.364, 0.383), CCT of 4508 K, and CRI of 89.7 is undoubtedly superior to that of the pc-WLED fabricated with a blue chip and YAG:Ce^3+^ phosphor. For further comparison, pc-WLED-2 (Fig. 7c) is fabricated similarly by using commercial red K_2_SiF_6_:Mn^4+^, green (Ba,Sr)_2_SiO_4_:Eu^2+^, and blue BAM:Eu^2+^ phosphors with a 395 nm n-UV LED chip. The CCT and CRI of pc-WLED-2 for K_2_SiF_6_:Mn^4+^ are 5850 K and 71.5, respectively, indicating that the CCT and CRI of pc-WLED-1 fabricated using CEGO are better than those of pc-WLED-2 fabricated using commercial red K_2_SiF_6_:Mn^4+^. Another pc-WLED fabricated by green (BaSr)_2_SiO_4_:Eu^2+^ and red K_2_SiF_6_:Mn^4+^ with a blue chip exhibits a lower CRI (55.2). The luminous efficiency of pc-WLED-1 for CEGO is 74.61 lm/W, which is obviously lower than that of pc-WLED-2 utilising commercial K_2_SiF_6_:Mn^4+^ (137.94 lm/W). In addition, the CIE coordinates of the fabricated warm pc-WLEDs in Fig. 7d and the illumination pictures in the insets predict that this red CEGO phosphor has good application in warm pc-WLEDs.

## Discussion

In summary, non-concentration quenching in a series of red CGGO:*x*Eu^3+^ (*x* = 0.1–1.0) solid-solution phosphors is studied in detail for the first time. All these compounds crystallise in the orthorhombic structure with space group Pna21 (No. 33). Due to the layer structure feature of the CGGO host, the longer nearest distance greatly reduces the possibility of the effective energy being captured by quenching centres, even at the highest concentration of Eu^3+^ (CEGO). This non-concentration quenching red CEGO phosphor could convert the n-UV and blue light excitations into highly efficient red light with CIE coordinates of (0.6517, 0.348), a high colour purity of 95.07% and an IQE of 94%. Remarkably, this CEGO phosphor with an orthorhombic structure exhibits a volumetric NTE (−5.06 × 10^−5^/°C) and has good thermal stability at a high temperature (250 °C, >90%) owing to its higher symmetry, which is even better than typical K_2_SiF_6_:Mn^4+^ and Y_2_O_3_:Eu^3+^ red phosphors in the high-temperature region (175–250 °C). Moreover, the fabricated n-UV-based pc-WLED with red CEGO, green (Ba,Sr)_2_SiO_4_:Eu^2+^ and blue BAM:Eu^2+^ phosphors achieves a high CRI (89.7) and low CCT (4508 K). The above results indicate that this novel, highly efficient, and thermally stable red phosphor is a superb candidate for the lighting field, opening a new perspective for the development of luminescent materials.

## Materials and methods

### Materials synthesis

Cs_3_Gd_1-*x*_Ge_3_O_9_:*x*Eu^3+^ (CGGO:*x*Eu^3+^) (*x* = 0–1) samples were synthetised *via* a high-temperature solid-state reaction process. The raw materials Cs_2_CO_3_ (99.99%), GeO_2_ (A.R.), Gd_2_O_3_ (99.99%) and Eu_2_O_3_ (A.R.) were mixed according to stoichiometric molar ratios, and ground thoroughly in an agate mortar with pestle for more than 20 min. Then, the mixture was transferred into aluminium oxide crucibles and sintered at 1000 °C for 8 h in air. After the furnace cooled down to room temperature naturally, the final phosphors were obtained after grounding again.

### Characterisation

Powder XRD was collected on a D8 Advance X-ray Diffractometer (Bruker AXS, Germany) at a scanning rate of 1° min^−1^ in the 2*θ* range from 10 to 100° utilising Cu Kα radiation (*λ* = 1.5418 Å) at room temperature. Temperature-dependent XRD was also performed by this instrument with a temperature controller. Rietveld refinements of XRD patterns were conducted by a general structure analysis system programme. DR spectra were recorded by a UV–vis–NIR spectrophotometer (Hitachi U-4100). PL excitation (PLE) and PL spectra were obtained by a fluorescence spectrophotometer equipped with a 150 W xenon lamp as the excitation source (Edinburgh Instruments FLSP-920). The thermal stability of the samples was evaluated by the same instrument with a temperature controller. PL decay curves were acquired using a Lecroy Wave Runner 6100 Digital Osilloscope (1 GHz) with a tunable laser (pulse width 4 ns; gate 50 ns) as the excitation (Contimuum Sunlite OPO). IQEs were collected on an absolute PL quantum yield measurement system (Hamamatsu photonics K.K., C9920-02 Japan) under 464 nm excitation wavelength. The electroluminescence (EL) performance of pc-WLED devices was measured by Starspec SSP6612.

### LED fabrication

pc-LED devices were fabricated by combining red CEGO, commercial blue BAM:Eu^2+^ and green (Ba,Sr)_2_SiO_4_:Eu^2+^ phosphors, coated on a 395 nm n-UV LED chip using transparent silicon resin (A:B = 1:1) as the binder. Commercially available red K_2_SiF_6_:Mn^4+^, blue BAM:Eu^2+^ and green (Ba,Sr)_2_SiO_4_:Eu^2+^ phosphors were used to fabricate pc-WLEDs for comparison.

## Supplementary information

Supplementary material
