# Supplementary material for: Thermally stable and highly efficient red-emitting Eu3+-doped Cs3GdGe3O9 phosphors for WLEDs: non-concentration quenching and negative thermal expansion
Source: Light Sci Appl. 2021 Feb 1;10:29. doi: 10.1038/s41377-021-00469-x (PMC7851390; doi:10.1038/s41377-021-00469-x)
Supplement: Supplementary file 1 — Supplementary material [file 41377_2021_469_MOESM1_ESM.pdf]

Supplementary Information for thermally stable and highly efficient red-emitting Eu^{3+}-doped $Cs_3GdGe_3O_9$ phosphors for WLEDs: non-concentration quenching and negative thermal expansion

Peipei Dang,[1,2] Guogang Li,[3,*] Xiaohan Yun,[3] Qianqian Zhang,[1,2] Dongjie Liu,[1,2] Hongzhou Lian,[1] Mengmeng Shang,[4] and Jun Lin[1,2,5,*]

[1] *State Key Laboratory of Rare Earth Resource Utilization, Changchun Institute of Applied Chemistry, Chinese Academy of Sciences, 130022 Changchun, China. E-mail: jlin@ciac.ac.cn*

[2] *University of Science and Technology of China, 230026 Hefei, China*

[3] *Engineering Research Center of Nano-Geomaterials of Ministry of Education, Faculty of Materials Science and Chemistry, China University of Geosciences, 430074 Wuhan, China. E-mail: ggli@cug.edu.cn*

[4] *School of Material Science and Engineering, Shandong University, 266071 Jinan, China*

[5] *School of Applied Physics and Materials, Wuyi University, 529020 Guangdong, China*

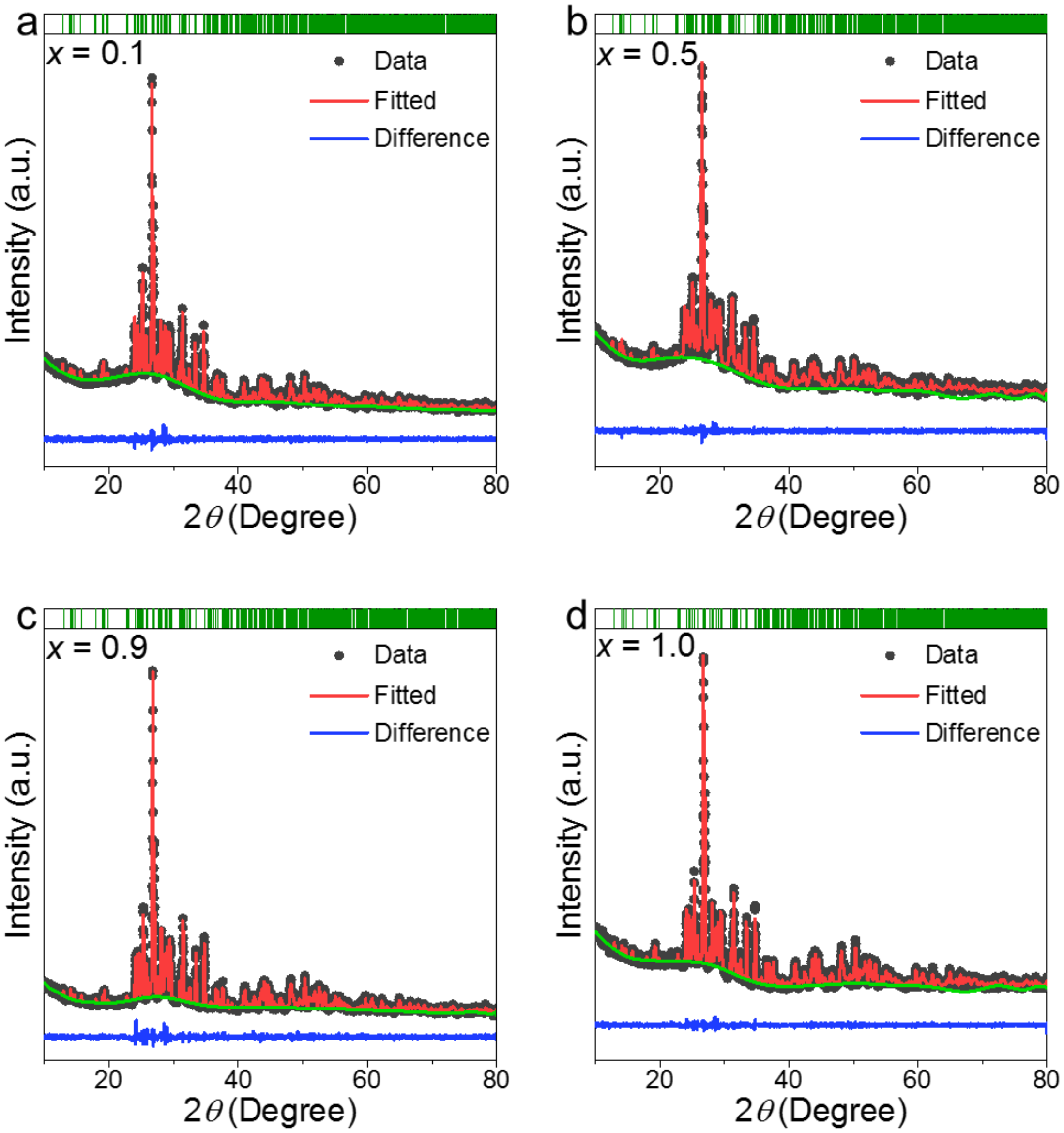

Figure S1. Rietveld refinement of CGGO:xEu^{3+} samples: (a) x = 0.1, (b) x = 0.5, (c) x = 0.9, and (d) x = 1.0.

Table S1. Refined structural data of CGGO:xEu^{3+} (x = 0.1-1.0) samples.

**Samples (x)**	**Cell parameters (Å)**	**Volume (Å^3)**	**R_{wp}, R_p, %, χ^2**
0.1	a = 13.8944(19)<br>b = 7.1665(10)<br>c = 12.7825(18)	1272.81(31)	3.95, 3.07, 1.465
0.2	a = 13.8990(9)<br>b = 7.1664(5)<br>c = 12.7882(8)	1273.79(14)	4.24, 3.16, 1.638
0.3	a = 13.9007(9)<br>b = 7.1692(6)<br>c = 12.7895(10)	1274.57(17)	3.67, 2.83, 1.305
0.4	a = 13.9054(11)<br>b = 7.1718(7)<br>c = 12.7924(11)	1275.74(20)	4.48, 3.37, 1.808
0.5	a = 13.9086(6)<br>b = 7.1738(4)<br>c = 12.7975(6)	1276.91(11)	4.01, 3.05, 1.681
0.6	a = 13.9158(18)<br>b = 7.1787(9)<br>c = 12.8005(17)	1278.73(29)	5.29, 3.98, 2.530
0.7	a = 13.9211(11)<br>b = 7.1773(7)<br>c = 12.8067(10)	1279.59(19)	4.45, 3.44, 1.967
0.8	a = 13.9258(9)<br>b = 7.1888(6)<br>c = 12.8142(8)	1282.83(16)	4.96, 3.66, 2.391
0.9	a = 13.9201(13)<br>b = 7.1902(8)<br>c = 12.8111(10)	1282.24(21)	5.49, 4.11, 2.433
1.0	a = 13.9190(17)<br>b = 7.1967(11)<br>c = 12.8074(19)	1282.93(31)	4.03, 3.08, 1.327

Table S2. The atom positions, fraction factors, and thermal vibration parameters of CGGO:$x$$Eu^{3+}$ (x = 0.1, 0.5, 0.9, 1.0) samples.

Atom	Site	*x*	*y*	*z*	Uiso (*100)	Occupancy
			***x* = 0.1**			
Gd1	*4a*	0.46505(6)	0.15900(7)	0.55151(1)	1.87	0.9123
Eu	*4a*	0.46505(6)	0.15900(7)	0.55151(1)	1.87	0.0877
Cs1	*4a*	0.32925(1)	-0.04253(8)	0.78876(2)	2.51	1.0065
Cs2A	*4a*	0.17322(2)	-0.05196(1)	0.44030(8)	4.01	0.9435
Cs2B	*4a*	0.13165(6)	-0.09602(7)	0.59251(2)	3.65	0.0610
Cs3A	*4a*	0.43814(8)	1.39610(2)	0.22371(4)	4.07	0.8545
Cs3B	*4a*	0.46415(6)	1.40286(9)	0.27827(5)	1.56	0.1455
Ge1	*4a*	0.22326(5)	0.38775(3)	0.59265(8)	2.21	1.0250
Ge2	*4a*	0.39958(4)	0.66596(5)	0.50614(1)	1.24	1.0088
Ge3	*4a*	0.40842(4)	0.89537(6)	0.30218(1)	0.88	0.9916
O1	*4a*	0.14740(5)	0.36683(3)	0.49511(2)	1.89	0.9625
O2	*4a*	0.27032(5)	0.23975(2)	0.62784(1)	0.80	1.0078
O3	*4a*	0.24975(3)	0.64549(1)	0.56351(1)	2.46	1.0176
O4	*4a*	0.45041(5)	0.52661(1)	0.49733(1)	2.16	0.9745
O5	*4a*	0.41797(9)	0.83925(5)	0.59048(1)	2.90	0.9707
O6	*4a*	0.35488(1)	0.74665(7)	0.35668(4)	1.80	0.9841
O7	*4a*	0.39808(8)	1.12055(4)	0.34879(8)	4.02	1.0212
O8	*4a*	0.52274(4)	0.80871(5)	0.20201(2)	2.83	0.9657
O9	*4a*	0.20245(4)	0.51375(2)	0.74396(2)	2.69	1.0016
			***x* = 0.5**			
Gd1	*4a*	0.45235(7)	0.15945(7)	0.53207(7)	1.78	0.5488
Eu	*4a*	0.45235(7)	0.15945(1)	0.53207(7)	1.78	0.4512
Cs1	*4a*	0.32254(1)	-0.05674(2)	0.79422(1)	3.50	1.0393
Cs2A	*4a*	0.17307(6)	-0.03514(4)	0.43739(6)	2.52	0.9241
Cs2B	*4a*	0.08602(4)	-0.13900(8)	0.58457(1)	1.63	0.0759

Cs3A	*4a*	0.43994(8)	1.40307(6)	0.22399(4)	3.24	0.8610
Cs3B	*4a*	0.47273(3)	1.40585(2)	0.25398(4)	3.39	0.1390
Ge1	*4a*	0.22519(8)	0.38431(3)	0.59377(9)	1.78	1.0484
Ge2	*4a*	0.39364(1)	0.66266(8)	0.50057(7)	0.90	1.0190
Ge3	*4a*	0.40566(5)	0.90045(8)	0.30028(4)	1.16	1.0706
O1	*4a*	0.16472(5)	0.37467(7)	0.51286(1)	1.82	0.9755
O2	*4a*	0.31476(9)	0.21288(2)	0.60273(3)	2.57	1.0949
O3	*4a*	0.25919(8)	0.65181(3)	0.54915(8)	2.25	1.0521
O4	*4a*	0.44427(9)	0.57920(6)	0.49066(9)	2.19	0.9211
O5	*4a*	0.41785(1)	0.82865(8)	0.57017(8)	3.42	0.9668
O6	*4a*	0.36280(1)	0.71160(2)	0.37380(2)	1.77	1.0000
O7	*4a*	0.44150(2)	1.08420(3)	0.36900(4)	2.56	1.0000
O8	*4a*	0.50040(1)	0.80550(2)	0.21810(2)	1.81	1.0000
O9	*4a*	0.19420(5)	0.45640(6)	0.72250(3)	2.68	1.0000
			***x* = 0.9**			
Gd1	*4a*	0.45602(1)	0.19773(9)	0.55999(8)	1.69	0.0977
Eu	*4a*	0.45602(1)	0.19773(9)	0.55999(8)	1.69	0.9023
Cs1	*4a*	0.32371(1)	-0.03361(3)	0.80229(1)	3.09	1.0480
Cs2A	*4a*	0.16892(7)	-0.03590(8)	0.43423(4)	2.51	0.8899
Cs2B	*4a*	0.13859(3)	-0.07869(2)	0.48331(7)	1.69	0.1101
Cs3A	*4a*	0.44155(5)	1.40396(7)	0.22372(8)	2.11	0.8743
Cs3B	*4a*	0.48393(4)	1.40660(5)	0.28112(5)	1.54	0.1257
Ge1	*4a*	0.22512(1)	0.38447(5)	0.59240(6)	1.43	0.9953
Ge2	*4a*	0.40110(2)	0.66067(1)	0.50071(1)	1.03	1.0323
Ge3	*4a*	0.40434(3)	0.89017(1)	0.30035(9)	1.80	0.9600
O1	*4a*	0.17521(8)	0.32826(7)	0.47858(6)	2.21	1.0413
O2	*4a*	0.31731(5)	0.18389(1)	0.60803(2)	2.17	0.9344
O3	*4a*	0.27993(8)	0.62583(4)	0.54114(4)	1.82	1.0594
O4	*4a*	0.51420(4)	0.54549(5)	0.50871(6)	1.97	0.9326

O5	*4a*	0.43501(8)	0.84064(9)	0.61081(8)	2.14	1.0199
O6	*4a*	0.37912(4)	0.67672(4)	0.38114(7)	2.37	0.9946
O7	*4a*	0.43679(2)	1.11413(3)	0.34296(1)	2.78	1.0248
O8	*4a*	0.50559(9)	0.79289(2)	0.21040(2)	2.40	0.9137
O9	*4a*	0.22413(8)	0.46125(9)	0.71185(9)	3.00	1.1122
			***x* = 1.0**			
Eu1	*4a*	0.48093(2)	0.15758(3)	0.56490(2)	0.93	1.0253
Cs1	*4a*	0.33258(1)	-0.03169(9)	0.80385(6)	2.65	1.0969
Cs2A	*4a*	0.16935(4)	-0.11759(3)	0.45799(3)	2.89	0.9406
Cs2B	*4a*	0.16290(1)	-0.14588(7)	0.47209(3)	2.89	0.0594
Cs3A	*4a*	0.43185(3)	1.38182(3)	0.24921(3)	3.12	0.8346
Cs3B	*4a*	0.36482(1)	1.33100(6)	0.22801(9)	3.12	0.1654
Ge1	*4a*	0.22469(7)	0.35981(4)	0.59532(7)	1.10	1.0464
Ge2	*4a*	0.39694(7)	0.65895(1)	0.50711(5)	1.04	1.0926
Ge3	*4a*	0.40125(6)	0.90722(5)	0.31118(9)	1.09	0.9590
O1	*4a*	0.12313(2)	0.38031(6)	0.52549(9)	1.94	1.0145
O2	*4a*	0.26781(4)	0.20260(3)	0.58789(1)	2.13	0.9746
O3	*4a*	0.26522(1)	0.62045(4)	0.53539(4)	2.46	1.0035
O4	*4a*	0.47657(3)	0.63215(1)	0.46998(9)	2.12	0.9490
O5	*4a*	0.41969(1)	0.80737(7)	0.63548(7)	2.28	1.0129
O6	*4a*	0.35067(6)	0.69023(8)	0.37426(4)	1.83	1.0732
O7	*4a*	0.42782(1)	1.18876(6)	0.33022(4)	2.10	1.0422
O8	*4a*	0.53791(2)	0.86762(6)	0.20565(1)	2.23	1.0229
O9	*4a*	0.22322(5)	0.39934(8)	0.74490(7)	2.43	1.0552

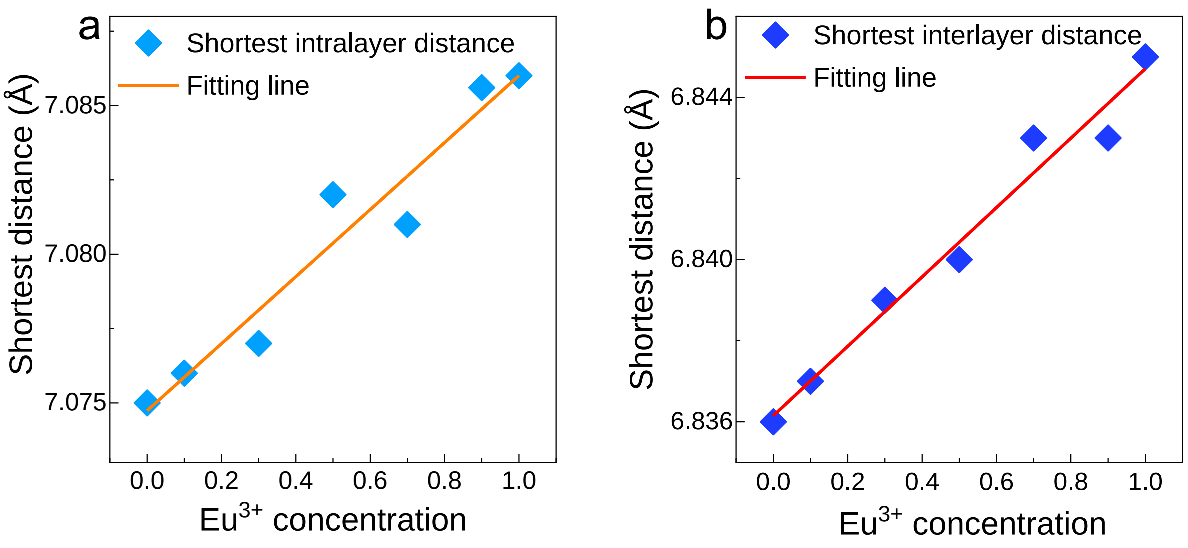

Figure S2. The shortest (a) intralayer distance and (b) interlayer distance between two Gd^{3+}/Eu^{3+} ions in CGGO:$x$$Eu^{3+}$ (x = 0-1.0) samples.

Table S3. CIE coordinates, IQE and color purity of CGGO:xEu^{3+} (x = 0.1-1.0) samples under 464 nm blue light excitation.

**Samples (x)**	**CIE x**	**CIE y**	**IQE**	**EQE**	**Color purity (%)**
0.1	0.6489	0.3508	0.124	0.021	94.28
0.2	0.6503	0.3493	0.190	0.030	94.67
0.3	0.6512	0.3485	0.365	0.040	94.93
0.4	0.6512	0.3484	0.386	0.039	94.93
0.5	0.6513	0.3484	0.419	0.075	94.95
0.6	0.6513	0.3484	0.432	0.068	94.95
0.7	0.6514	0.3483	0.696	0.109	94.98
0.8	0.6516	0.3481	0.763	0.078	95.04
0.9	0.6517	0.348	0.857	0.077	95.07
1.0	0.6517	0.348	0.940	0.085	95.07
Y_2O_3:Eu^{3+}	0.6435	0.3551	0.080	-	91.83
K_2SiF_6:Mn^{4+}	0.6905	0.3093	0.948	0.452	94.63

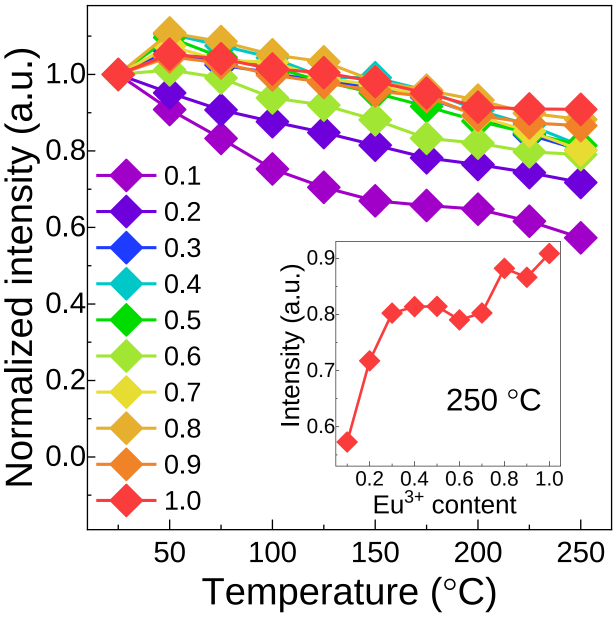

Figure S3. Temperature-dependent PL intensity of CGGO:xEu^{3+} (x = 0.1-1.0) samples.

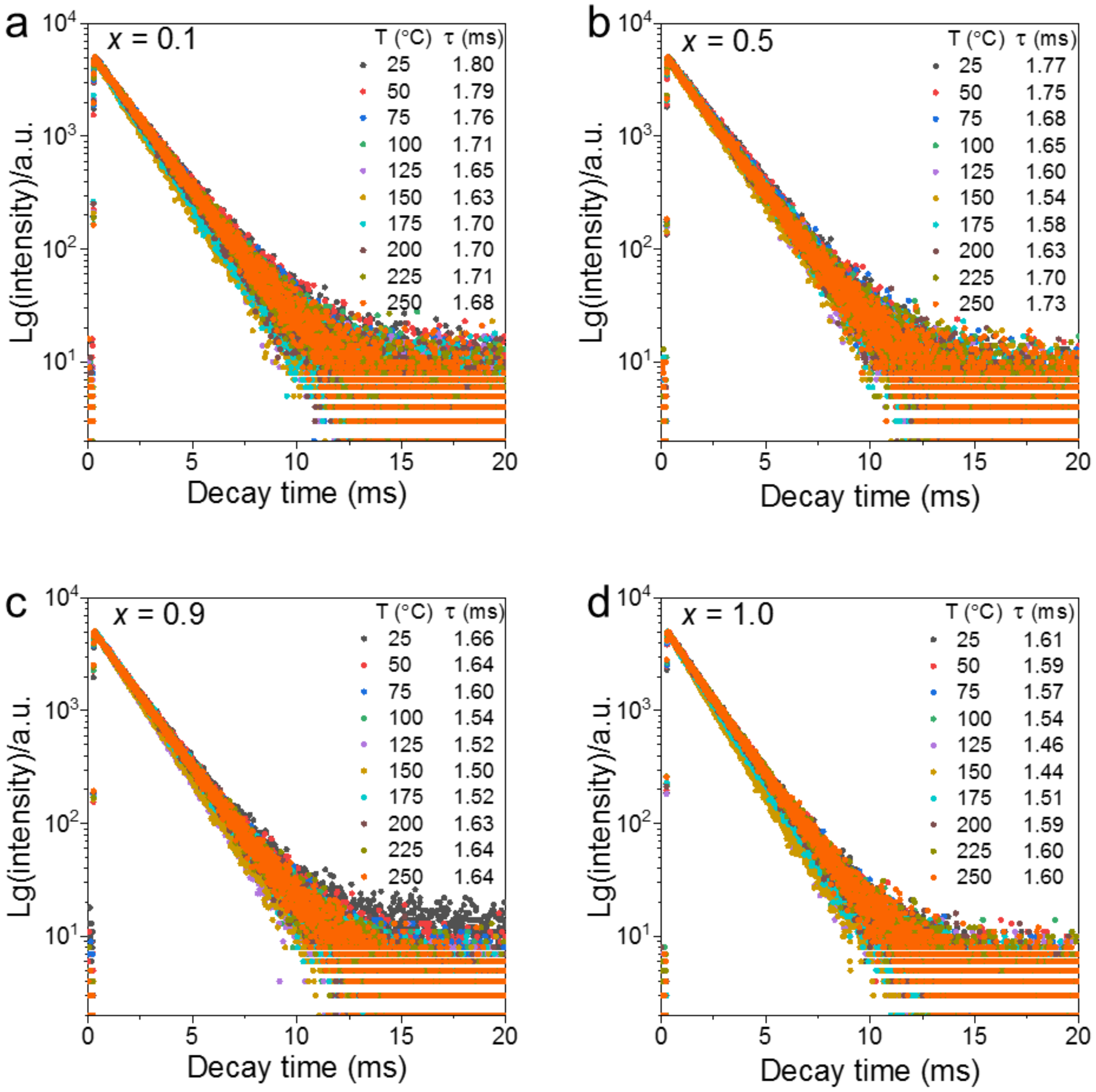

Figure S4. PL decay lifetimes of CGGO:*x*Eu^{3+} samples: (a) *x* = 0.1, (b) *x* = 0.5, (c) *x* = 0.9, and (d) *x* = 1.0.

Table S4. CIE coordinates and color purity of CEGO at different temperatures from 25 to 250 °C.

**T (°C)**	**CIE *x***	**CIE *y***	**Color purity (%)**
25	0.6517	0.348	95.07
50	0.6519	0.3478	95.12
75	0.6516	0.348	95.04
100	0.6511	0.3486	94.90
125	0.6504	0.3492	94.70
150	0.6496	0.3500	94.47
175	0.6488	0.3508	94.25
200	0.6477	0.3518	93.94
225	0.6464	0.3531	93.57
250	0.6446	0.3548	93.07

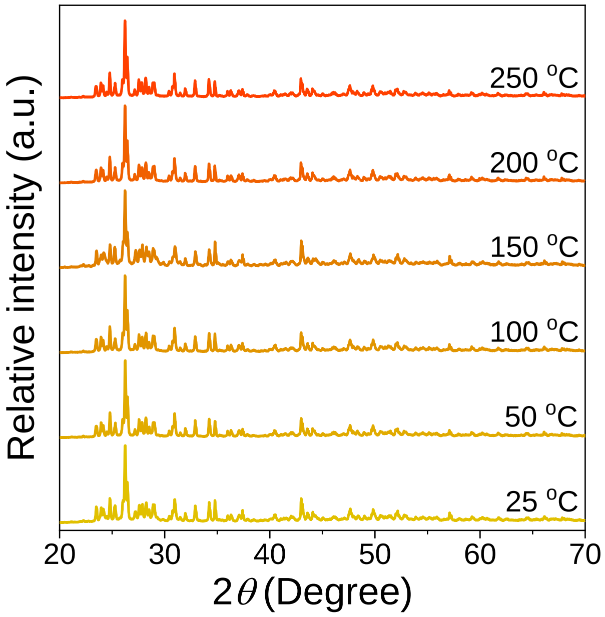

Figure S5. XRD patterns of CEGO sample at different temperatures (25-250 °C).

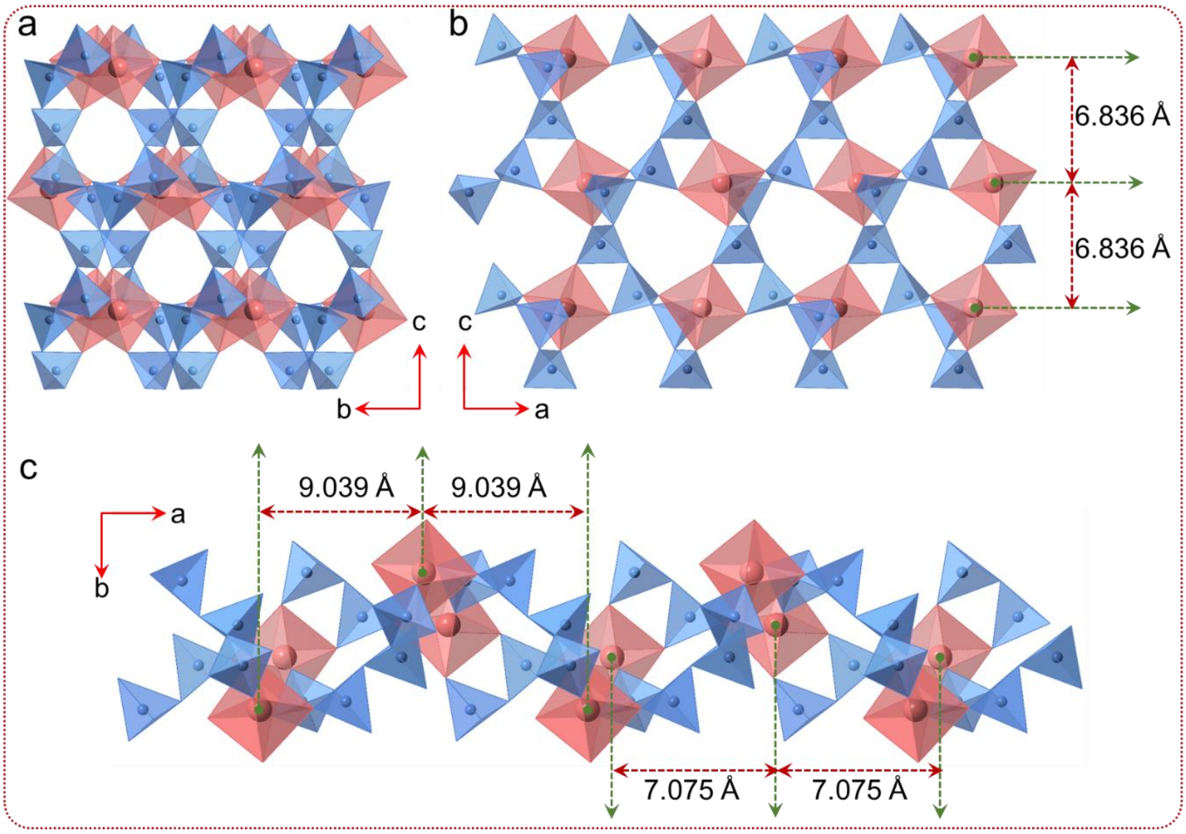

Figure S6. (a) Layer-distributed Gd sites along the c-axis direction, (b) the distance of interlayer Gd sites and (c) the distance of intralayer Gd sites.

Table S5. Comparison of excitation position, IQE and thermal stability ($I_{200\,°C}/I_{25\,°C}$) for $Cs_3EuGe_3O_9$ and other reported Eu^{3+}-doped phosphors and typical commercial red phosphors.

Phosphors	λ_{ex}	IQE	$I_{200\,°C}/I_{25\,°C}$	Ref.
$Ba_6Gd_2Ti_4O_{17}:Eu^{3+}$	394 nm	24%	~36%	1
$Ca_4EuO(BO_3)_3:Eu^{3+}$	395 nm	98%	~50%	2
$Ca_3Eu_2B_4O_{12}$	393 nm	95.6%	~65%	3
$LaSc_3(BO_3)_4:Eu^{3+}$	393 nm	89%	~73%	4
$Y_2O_3:Eu^{3+}$	280 nm	83%	~75%	2
$Y_2Mg_2Al_2Si_2O_{12}:Eu^{3+}$	393 nm	60.5%	~80%	5
$Li_6CaLa_2Nb_2O_{12}:Eu^{3+}$	393 nm	51%	~80%	6
$K_2SiF_6:Mn^{4+}$	460 nm	95%	~82%	7
$K_5Eu(P_2O_7)_2$	377 nm	81%	~87%	8
$Cs_3EuGe_3O_9$	464 nm	94%	~90%	this work

References

1 Li, J. H. *et al.* Layered structure produced nonconcentration quenching in a novel Eu^{3+}-doped phosphor. *ACS Appl. Mater. Interfaces* **10**, 41479-41486 (2018).

2 Chen, Z. *et al.* Tunable yellow-red photoluminescence and persistent afterglow in phosphors $Ca_4LaO(BO_3)_3:Eu^{3+}$ and $Ca_4EuO(BO_3)_3$. *Inorg. Chem.* **55**, 11249-11257 (2016).

3 Li, G. H. *et al.* The non-concentration-quenching phosphor $Ca_3Eu_2B_4O_{12}$ for WLED application. *Inorg. Chem.* **59**, 3894-3904 (2020).

4 Yang, N. *et al.* Delayed concentration quenching of luminescence caused by Eu^{3+}-induced phase transition in $LaSc_3(BO_3)_4$. *Chem. Mater.* **32**, 6958-6967 (2020).

5 Zhang, X. T. *et al.* Study on the local structure and luminescence properties of a $Y_2Mg_2Al_2Si_2O_{12}:Eu^{3+}$ red phosphor for white-light-emitting diodes. *Inorg. Chem.* **59**, 9927-9937 (2020).

6 Du, P. P. *et al.* Sol-gel processing of Eu^{3+} doped $Li_6CaLa_2Nb_2O_{12}$ garnet for efficient and thermally stable red luminescence under near-ultraviolet/blue light excitation. *Chem. Eng. J.* **375**, 121937 (2019).

7 Hou, Z. Y. *et al.* A green synthetic route to the highly efficient $K_2SiF_6:Mn^{4+}$ narrow-band red phosphor for warm white light-emitting diodes. *J. Mater. Chem. C* **6**, 2741-2746 (2018).

8 Zhao, D. *et al.* Non-concentration quenching, good thermal stability and high quantum efficiency of $K_5Y(P_2O_7)_2:Eu^{3+}/Tb^{3+}$ phosphors with a novel two-dimensional layer structure. *J. Mater. Chem. C* **7**, 14264-14274 (2019).

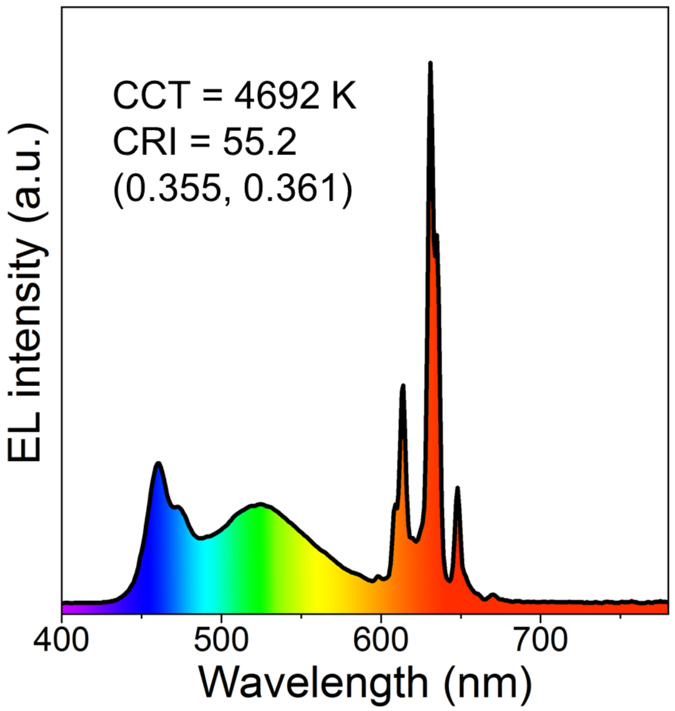

Figure S7. EL spectrum of pc-WLED device (blue chip + green $(Ba,Sr)_2SiO_4:Eu^{2+}$ + red $K_2SiF_6:Mn^{4+}$).
